# Automatic Detection of Flavescence Dorée Symptoms Across White Grapevine Varieties Using Deep Learning

**DOI:** 10.3389/frai.2020.564878

**Published:** 2020-11-30

**Authors:** Justine Boulent, Pierre-Luc St-Charles, Samuel Foucher, Jérome Théau

**Affiliations:** ^1^ Department of Applied Geomatics, Université de Sherbrooke, Sherbrooke, QC, Canada; ^2^ Computer Research Institute of Montréal, Montréal, QC, Canada; ^3^ Quebec Centre for Biodiversity Science (QCBS), Montréal, QC, Canada; ^4^ Applied Machine Learning Research Team, Mila, Montréal, QC, Canada

**Keywords:** precision viticulture, smart farming, plant diseases detection, Flavescence dorée, grapevine yellows, convolutional neural networks, fully convolutional networks, explainable artificial intelligence

## Abstract

*Flavescence dorée* (FD) is a grapevine disease caused by phytoplasmas and transmitted by leafhoppers that has been spreading in European vineyards despite significant efforts to control it. In this study, we aim to develop a model for the automatic detection of FD-like symptoms (which encompass other grapevine yellows symptoms). The concept is to detect likely FD-affected grapevines so that samples can be removed for FD laboratory identification, followed by uprooting if they test positive, all to be conducted quickly and without omission, thus avoiding further contamination in the fields. Developing FD-like symptoms detection models is not simple, as it requires dealing with the complexity of field conditions and FD symptoms’ expression. To address these challenges, we use deep learning, which has already been proven effective in similar contexts. More specifically, we train a Convolutional Neural Network on image patches, and convert it into a Fully Convolutional Network to perform inference. As a result, we obtain a coarse segmentation of the likely FD-affected areas while having only trained a classifier, which is less demanding in terms of annotations. We evaluate the performance of our model trained on a white grape variety, Chardonnay, across five other grape varieties with varying FD symptoms expressions. Of the two largest test datasets, the true positive rate for Chardonnay reaches 98.48% whereas for Ugni-Blanc it drops to 8.3%, underlining the need for a multi-varietal training dataset to capture the diversity of FD symptoms. To obtain more transparent results and to better understand the model’s sensitivity, we investigate its behavior using two visualization techniques, Guided Gradient-weighted Class Activation Mapping and the Uniform Manifold Approximation and Projection. Such techniques lead to a more comprehensive analysis with greater reliability, which is essential for in-field applications, and more broadly, for all applications impacting humans and the environment.

## Introduction

*Flavescence dorée* (FD) is a grapevine disease raising serious concern in Europe. This disease is caused by several phytoplasmas classified according to their ribosomal DNA (16SrV subgroup C and D) (Filippin et al., 2009) and grouped under the temporary name of *Candidatus Phytoplasma vitis* (Firrao et al., 2004). The transmission of those phytoplasmas is mediated by infected leafhopper *Scaphoideus titanus* which transmit the disease when feeding on vine leaves. This leafhopper, native to North America, was first observed in France in 1958 (Bonfils and Schvester, 1960). Adult *S. titanus* have a limited dispersal distance, reaching 25–30 m, although winds can cause passive dispersal over larger areas. However, the long distance spread is mainly caused by human activities, such as the trade of plant infested material (Chuche and Thiery, 2014). Despite a quarantine status and mandatory monitoring practices, FD is still spreading in Europe (Fanjul, 2017). FD symptoms appear gradually over the summer with a peak of expression at the end of August/September. Contaminated plants show a drooping appearance caused by a lack of lignification. Infected leaves roll up and change color: yellowing for white grape varieties and reddening for red grape varieties. FD causes the death of inflorescences and of berries (Chuche and Thiery, 2014). Symptoms expressed by an FD-infected plant are the same as those expressed by *Bois noir*, another disease caused by phytoplasmas. Both diseases are gathered under the term of “grapevine yellows symptoms.” To distinguish between them, laboratory analyses are necessary (Chuche and Thiery, 2014). Over the years, FD-contaminated plants will die or become less and less productive. In any case, they constitute an eventual source of contamination for the surrounding vineyards. In France, the uprooting of contaminated grapevines is mandatory. When 20% of the grapevines in a parcel are contaminated, the whole parcel must be uprooted (Ministère Français de l’Agriculture, 2013). Winegrowers are thus exposed to significant economic losses. Since there is no control method for phytoplasma and no treatment to cure an infected plant, the disease management has focused on the FD vector: the leafhopper. In some at-risk areas in France, Italy and Switzerland, insecticide treatments are mandatory to limit the number of leafhoppers (Chuche and Thiery, 2014).

Another strategy complementary to vector control has been investigated by several research teams: the automatic detection of FD symptoms in the field. This approach is based on the idea that by quickly locating all the grapevines that might be contaminated with FD, winegrowers are able to eliminate all of the at-risk plant material. Winegrowers already scout their fields, but due to a lack of time or of manpower, this tedious task is not always carried out systematically on the entire vineyard. With a camera on a drone, a tractor or a ground robot, a complete search could be carried out in a relatively short time. The winegrower or agronomist would then only have to check the suspect grapevines spotted by the detection tool and take appropriate actions. To develop such an automatic detection tool, AL-Saddik et al. (2017) opted for non-imaging and proximal hyperspectral analyses to find the optimal wavelengths to identify FD. Using several spectral measurements and considering grape variety and symptom intensity, they developed specific vegetation indices for FD detection. These indices resulted in classification accuracies of more than 90% on leaf-scale spectral signatures. Albetis et al. (2018) used multispectral drone images (combining Red-Green-Blue, or RGB, and Near-Infrared, or NIR) acquired on seven red grape varieties. From these images, 24 variables such as vegetation indices and biophysical parameters were computed and used to differentiate two study cases: 1) FD and wood diseases (Esca and Black Dead Arm) from asymptomatic grapevines, and 2) FD from wood diseases. Results were promising in the first study case, but more mixed in the second.

The processing of in-field images highlighted another challenge: the management of soil and shadow mixels, which are significant sources of misclassification. Cruz et al. (2019) used Convolutional Neural Networks (CNNs) on proximal RGB images to detect grapevine yellowing symptoms on a red grape variety, with particular emphasis on differentiating them from leafroll and *Stictocephala biosonia* symptoms. Their model achieved 98.96% sensitivity and 99.40% specificity. However, the images they used were collected under controlled conditions, which would not be suitable for an in-field automated detection tool. Nevertheless, numerous studies have shown the relevance of using CNNs to analyze in-field images on various crops (DeChant et al., 2017; Fuentes et al., 2018; Boulent et al., 2019b). Despite the difficulties in analyzing field images due to complex background, leaf entanglements and shadows, CNNs are robust enough to overcome those issues and find discriminating features to identify several diseases on different crops.

CNNs provide a modeling approach that is part of deep learning methods. CNNs consist of neural networks that can be trained to fit their internal parameters for a specific task based on a large amount of training data. More specifically, CNNs are primarily a set of convolutional and non-linear layers that extract hierarchical representations to solve a task defined through a loss function. For more detailed information on deep learning and CNNs, please refer to LeCun et al. (2015) and Goodfellow et al. (2016). The deployment of CNNs has led to great progress in several vision-related applications, particularly in image content classification.

Although FD is a complex disease whose symptoms are not easy to detect, we hypothesize that CNNs can tackle this challenging task. Several confusion factors can be expected: 1) other phytosanitary problems, such as downy mildew or deficiencies, can be expressed through similar symptoms, especially on white grape varieties (e.g., yellowing); 2) a combination of health issues can occur on the same leaves, particularly at the end of the season, which complicates the visual identification of FD; 3) the symptoms of FD can be expressed differently between varieties beyond the main two grape families (red and white) through variations in curling or coloring for example(AL-Saddik et al., 2017; Albetis et al., 2018); and 4) FD symptoms also seem to fluctuate from one year to the next, even for the same grape variety (Albetis et al., 2018).

The objective of this study is to achieve the automatic pre-identification of FD symptoms in several white grape varieties from images taken in the field. The term “pre-identification” refers to the fact that a laboratory analysis must be performed to ensure that a grapevine is indeed contaminated with FD. Our level of detection is downstream: the idea is to visually detect FD-like symptoms. Therefore, in this study, any mention of FD detection refers to this notion of visual “pre-identification.” The multi-varietal dimension is studied here because, for professional use, an automatic detection tool would be of real interest only if it could be used on several grape varieties. Acquiring images of all the targeted grape varieties would be expensive and very challenging. However, the symptoms, even within the same color family, can be quite different. So, could a model trained on one grape variety be effective on other grape varieties? To answer this question, we train a classification model on Chardonnay images and transform it into a Fully Convolutional Network (FCN, Shelhamer et al., 2017). We then test it on several independent datasets originating from different sources. To provide greater transparency to our model and its results, we use visualization techniques to understand which image features the model uses for its predictions. This also helps us understand the differences in precision obtained on the different grape varieties. By identifying the strengths and weaknesses of our model (along with ways to improve its robustness and reliability) we take one more step toward the development of a professional tool.

## Material and Methods

### Method Overview

**FIGURE 1:**
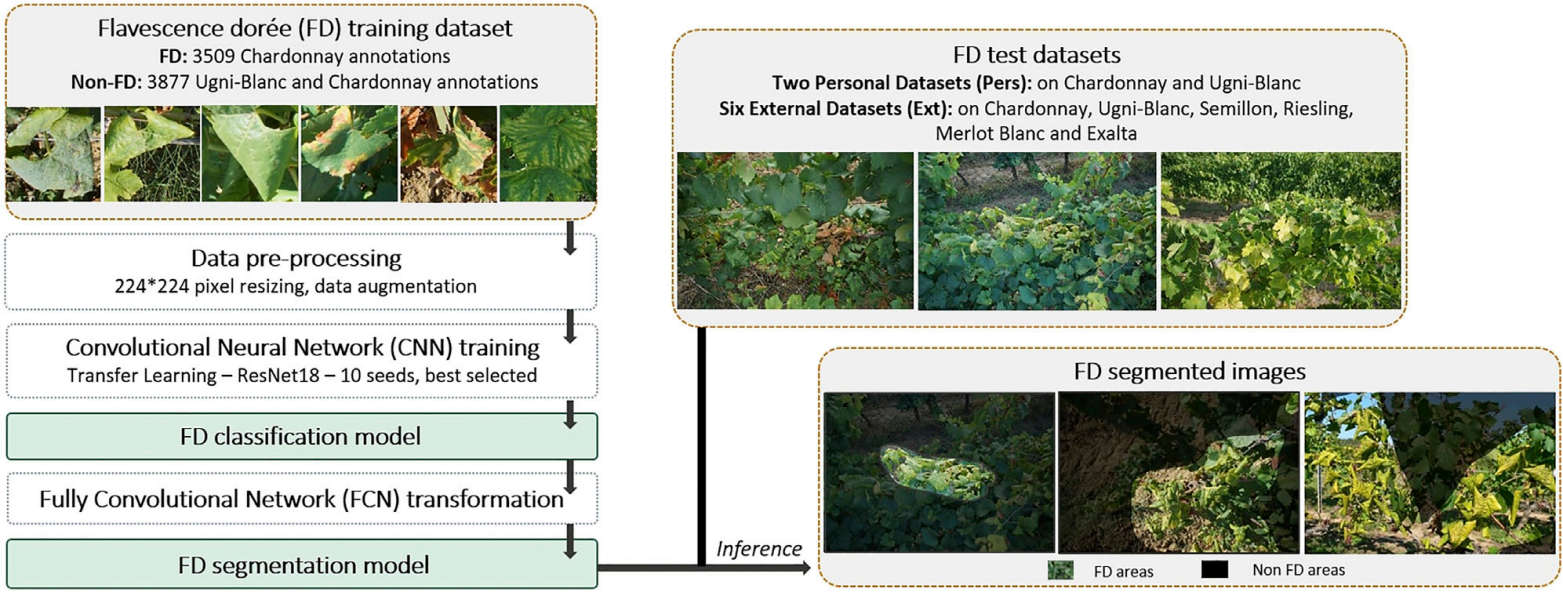
Processing setup overview.

To provide greater clarity, this preliminary section presents an overview of the processing steps performed in this study (Figure 1). To develop a tool to automatically detect FD symptoms, we first trained a CNN-based classifier. As an output, this model indicates whether or not there are FD-like symptoms in an input image. As a way to help the model’s training, we ensure that it targets elements of interest in the grapevines by annotating portions of images (mostly leaves). In the FD class of the training dataset, there are only Chardonnay leaves. The training process follows a classical approach: a ResNet18 is pre-trained on ImageNet and then completely fine-tuned with the FD training dataset. In fact, as our training dataset is quite small, it is advantageous to use weights from a prior training on a large dataset to retrieve the learned features as a starting point for our model training (Tajbakhsh et al., 2016; Tan et al., 2018).

The image-level binary classifier output (e.g., a unique FD or Non FD predicted label) is not the most suitable for our application. Indeed, a rough localization of the affected regions in the analyzed images would be much more appropriate. It would offer a more complete analysis while targeting suspect areas, bringing more transparency to the results and thus meeting the needs of the users. To achieve this rough localization, we opted to transform our classifier into an FCN. This transformation is simple in practice and it does not require any retraining to generate coarse class segmentation maps during inference (Shelhamer et al., 2017).

To test our model, we use several FD image datasets. Two are from personal acquisitions (*Pers.* datasets). These have the advantage of being quite large (153 images for the one on Chardonnay and 155 for the one on Ugni-Blanc) and they have a certain independence from the training dataset, as the images come from separate plots. However, they allow a possible bias: the images used for training and testing were taken during the same year, by the same team, and with similar cameras. To address this issue, we introduce a dataset from an external source (*Ext.* datasets), provided by FREDON (Fédération Régionale de Défense contre les Organismes Nuisibles) Aquitaine. Although small in size (34 images), it is of significant value, as it ensures that our model can work on images from completely independent sources. Furthermore, it also provides an opportunity to evaluate our model on several white grape varieties.

### Datasets

#### Image Collection Process

To train and test the FD pre-identification model, we use RGB images captured with standard cameras. The training images were taken in 2018, in France, in the Cognac ($45^{\circ }$41′24″″N, $0^{\circ }$19′12″W) and Limoux ($43^{\circ }$2′60″N, $2^{\circ }$12′36″E) regions. The data acquisition was carried out from mid-August to mid-September, allowing the recording of varied FD symptom intensities. During the scouting, we mainly found FD-infected grapevines in Chardonnay plots, hence our constraint to focus on this grape variety for the FD class. We also encountered combinations of FD symptoms with symptoms of downy mildew, pest damages and phytosanitary treatment residues. Thus, even though our training dataset for the FD class is single-varietal, it contains some diversity in the symptoms’ expression. During our scouting, we also encountered other plant health problems in Chardonnay and Ugni-Blanc plots: downy mildew, Esca, Black Dead Arm, damage caused by acarians, mineral deficiencies (in magnesium, potassium, manganese and iron) as well as burns due to pesticides. Two plots were selected as acquisition sites for the test datasets (referred to as *Pers.* datasets for “Personal” acquisition): one of Ugni-Blanc and one of Chardonnay. Thus, the images used to train and test the model came from separate plots.

Image acquisition was split based on two approaches: some images were captured using a hand-held camera, while others were captured from a camera mounted on a two-meter pole. The latter approach allowed a wider view of the grapevines. Due to the changing vineyard structure (inter-row distances, row heights, trimming type), it was not possible to define fixed acquisition parameters. The distance between the camera and the foliage as well as the acquisition angle thus vary from one photo to another. Hence, the photographs have slightly different spatial focus and resolution. No restrictions in terms of weather conditions were established, and the only requirement for acquisition was that the leaves had to be dry. During the acquisitions, images were taken from both shaded and sunny rows, resulting in varying light exposures.

**FIGURE 2:**
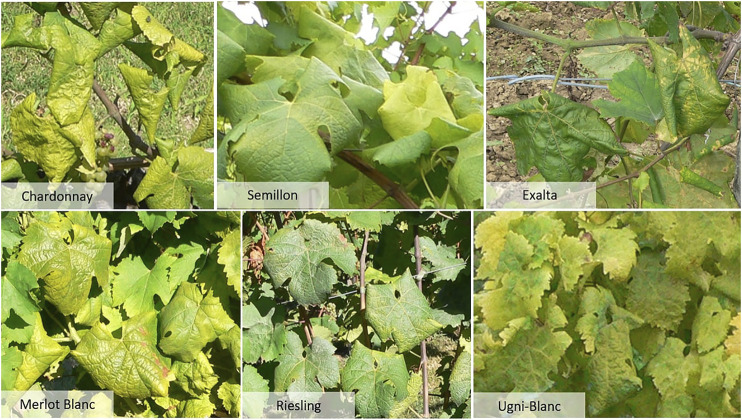
Illustration of Flavescence dorée symptoms for the different grape varieties found in the *Ext.* test dataset. Source: D. Vergnes, FREDON Aquitaine.

The set of 34 images prepared by a FREDON Aquitaine technician were reserved for testing.^1^ This set covers six grape varieties, with 3 to 12 images per variety: Chardonnay, Ugni-Blanc, Merlot Blanc, Riesling, Exalta and Semillon. All the images contain symptomatic FD grapevines. Figure 2 shows the typical expressions of the different grape varieties in this external (*Ext.*) dataset. Here, Chardonnay expresses a significant rolling with yellowing of a shade that differs from that of the training set. Exalta has rolled leaves with a withered aspect, sometimes with yellowing veins. Similar coloring is found on some Semillon leaves. Merlot Blanc and Riesling leaves show medium curling and yellowing. As for the Ugni-Blanc, it has little curling but significant coloration, sometimes with yellowing of the veins. Such variations in expression suggest generalization difficulties for our Chardonnay model on grape varieties expressing little curling. Although small in size, this test dataset is particularly relevant for the evaluation of our model because it was acquired by researchers not involved in this study. We know neither the camera model nor the year of acquisition, and had no control over the acquisition conditions. It therefore constitutes a realistic robustness test.

Table 1 summarises the information about the datasets used to train the CNN classifier and to evaluate the FCN model.

**TABLE 1:** Summary of datasets used to train the CNN classifier and to evaluate the FCN model.

Dataset name	Grapevine varieties	Number of images
CNN training dataset	FD class: Chardonnay, non FD class: Chardonnay, Ugni-Blanc	Total: 894^a^
FCN evaluation datasets		
*Pers. Chardonnay* test	Chardonnay	FD class: 66
		Non FD class: 87
		Total: 153
*Ext. Chardonnay* test	Chardonnay	FD class: 5
		Non FD class: None
		Total: 5
*Pers. Ugni-Blanc* test	Ugni-Blanc	FD class: 12
		Non FD class: 143
		Total: 155
*Ext. Other Grapevine Varieties* test	Ugni-Blanc, Exalta, Merlot Blanc, Semillon, Riesling	FD class: 29
		Non FD class: None
		Total: 29

a As we extracted samples for both classes in some images, we only indicate the total images’ count for the CNN Training dataset.

#### Data Preparation

##### Taxonomy Definition and Annotation

A binary taxonomy was chosen for our classification model: FD versus Non FD. This choice was made primarily because it simplifies the annotation step. While several diseases were present in the surveyed plots, and these sometimes appeared in combinations, we do not have the expertise required to identify all the diseases we observed. The binary taxonomy therefore allows us to put everything that does not look like FD into a generic “Non FD” class. This class contains healthy grapevines, but also includes weeds, soil, sky and trellising elements. On the other hand, the FD class only contains leaves with FD-like symptoms, although no laboratory tests were conducted to confirm that FD was truly present.

**FIGURE 3:**
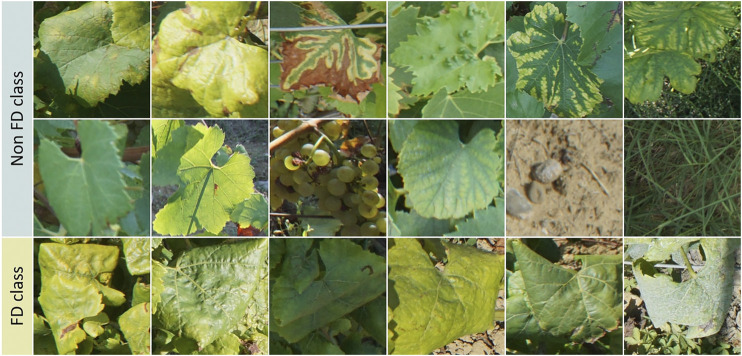
Examples of samples from the CNN training dataset for FD and Non-FD classes.

To train the classifier, we manually extracted diverse samples from the captured images. For the plant elements, a similar unit was used throughout the annotation: the leaf or the bunch of grapes. We identified the minimal enclosing square for each image region of interest. Around 7,300 annotations were generated to train the classifier, with 3,509 for the FD class and 3,877 for the Non-FD class. Some samples within these classes are shown in Figure 3. Before each training session, the samples are randomly separated into training samples (85%, or 6 279) and validation samples (15%, or 1 107). In order to account for this random split in our evaluation results, we run a series of 10 independent experiments while shuffling these sample sets.

To test the classification model post-FCN conversion, we manually generate segmentation maps by coarsely delimiting the image regions potentially contaminated by FD. Segmentation maps were also manually generated on 15 images from the training set to help choose the best model out of our 10 experiments.

##### Image preprocessing

The samples used for training our binary classifier are resized to 224×224 pixels. Since we use transfer learning based on ImageNet weights, we normalize the images using the mean and standard deviation values given for the RGB channels on the PyTorch website.^2^ To increase the model’s robustness to geometric and dynamic effects and to add diversity to our images, the following data augmentation operations are applied on the images during training: image rotations (probability: 0.6), distortions (probability: 0.6), flips (probability: 0.8) and changes in brightness (probability: 0.6). These transformations are applied randomly to each sample using Augmentor.^3^ For FCN inference, only the normalization was reapplied.

### Methods

#### Training

As mentioned earlier, the FD pre-identification model is based on a fine-tuned ResNet-18 architecture (He et al., 2016) pre-trained on ImageNet. This architecture is well-known and performed well in our previous experiments despite its simple nature (Boulent et al., 2019a). The hyperparameter values are fixed based on several trials that involved the learning rate definition method proposed by Smith (2018) implemented in the FastAi library.^4^ The values of the hyperparameters are provided in Table 2. The framework used to fine-tune and test the model is available on GitHub.^5^

**TABLE 2:** Hyperparameters used to trained the ResNet-18 classifier.

Hyperparameter name	Hyperparameter value
Learning rate	1e−04
Learning rate scheduler	Step size: 5, Gamma: 0.7
Optimizer	Adam
Loss function	Cross entropy
Batch size	64
Epochs	15

#### Inference

Once trained, our image classifiers were converted to FCN models for semantic segmentation following the methodology detailed by (Shelhamer et al., 2017). By replacing the fully connected layer with a 1 × 1 convolutional layer, a coarse segmentation map can be produced as the result of inference. Such limited resolution is caused by the use of pooling layers as well as the stride and padding values of the convolution layers. As a result, the output segmentation map is much smaller than the original image. To overlay with the original image, we resize this result to the input image’s size using bilinear interpolation.

We select the best CNN model out of our 10 experiments, using both validation accuracy and a visual quality assessment of the output segmentation maps on a handful of samples. The selected model is then evaluated on the four test sets: the two Chardonnay (*Pers.* and *Ext.*) as well as two other white grape varieties (*Pers.* and *Ext.*). Three metrics are used for performance evaluation: the True Positive Rate (TPR, Eq. 1) and the False Positive Rate (FPR, Eq. 2) to evaluate the quantity of both the correct and the incorrect detections generated by the model, and the Intersection over Union (IoU, Eq. 3), which provides a balanced overall look at the quality of the segmentation maps produced by the model. A Positive (P) sample is qualified as a “True” Positive (TP) if there was an intersection between the prediction and the annotation for the FD areas. A Negative (N) sample is qualified as a “True” Negative (TN) if the entire image was correctly predicted as Non-FD. The TPR was therefore more restrictive than a simple classification: it ensured that the detection was due to an FD area. Since for FD pre-identification, a false negative is more costly than a false positive, the decision threshold for the FD class has been set at 40% instead of the 50% usually used in binary classification.

$$TPR=\frac{TP}{P}\times 100,$$ (1)

where TP is the number of True Positive images and *P* is the number of Positive images;

$$FPR=\frac{FP}{N}\times 100,$$ (2)

where FP is the number of False Positives images and *N* is the total number Negatives of images; and

$$IoU_{AB}=\frac{A\cap B}{A\cup B},$$ (3)

where A is the set of all FD pixels predicted by the model and B is the set of all pixels labeled as FD.

## Results

**FIGURE 4:**
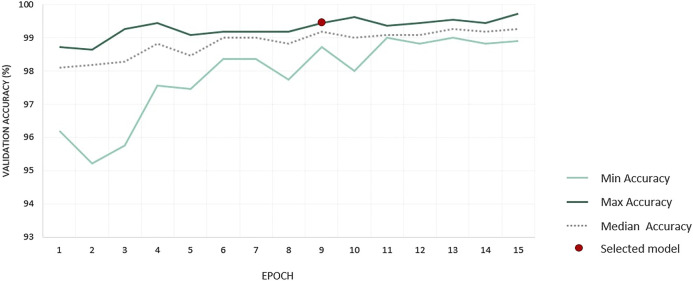
Summary of accuracy values obtained on the validation dataset for the 10 runs.

During the fine tuning, all models converged quickly (see Figure 4). By the end of the first epoch, the accuracies ranged from 96.2% to 98.7% on the validation dataset for all 10 runs. From the 11th epoch, accuracy values became very close, with a difference between the minimum and maximum accuracies of only 0.59% on average for the last 4 epochs. We transformed several of the obtained models with the highest precision values into FCNs, and used our validation dataset of 15 images to evaluate them. We decided to select the model with the highest precision value at the 9th epoch.

### Inference on Chardonnay Datasets

For both the Chardonnay test datasets, the TPR values are very high: 98.5% for the *Pers. Chardonnay* dataset (i.e., 65 TP for 66 P), 100% for *Ext. Chardonnay* dataset (i.e. 5 TP for 5 P). There is a low number of false alarms for the *Pers. Chardonnay* dataset, with an FPR of 1.15% (i.e., 1 FP for 87 N). These results highlight the detection power of the trained model. Next, the IoU values provide insight on the quality of the segmentation maps. The average IoU value is 0.53 for the *Pers. Chardonnay* dataset (with values ranging from 0.14 to 0.75), and 0.39 for the *Ext. Chardonnay* dataset (with values ranging from 0.26 to 0.6) (Table 3). Such values indicate a rather coarse segmentation quality. This was as expected, due to the simple classifier’s conversion to an FCN and its lack of advanced upsampling.

**TABLE 3:** Summary of inference results on both Chardonnay datasets, presenting the True Positive Rate (TPR), False Positive Rate (FPR), mean, min and max values of Intersection over Union (IoU).

Test Dataset Name	Number of images	TPR	FPR	IoU mean	IoU min^a^	IoU max
*Pers. Chardonnay*	FD class: 66 Non FD class: 87 Total: 153	98.48% (65 TP/66 P)	1.15% 1 FP/87 N	0.53	0.14	0.75
*Ext. Chardonnay*	FD class: 5 Non FD class: none Total: 5	100% (5 TP / 5 P)	N/A	0.39	0.26	0.6

Note: TP, True Positive; P, Positives; FP, False Positive; N, Negatives.

a IoU min, considering only the True Positive predictions.

**FIGURE 5:**
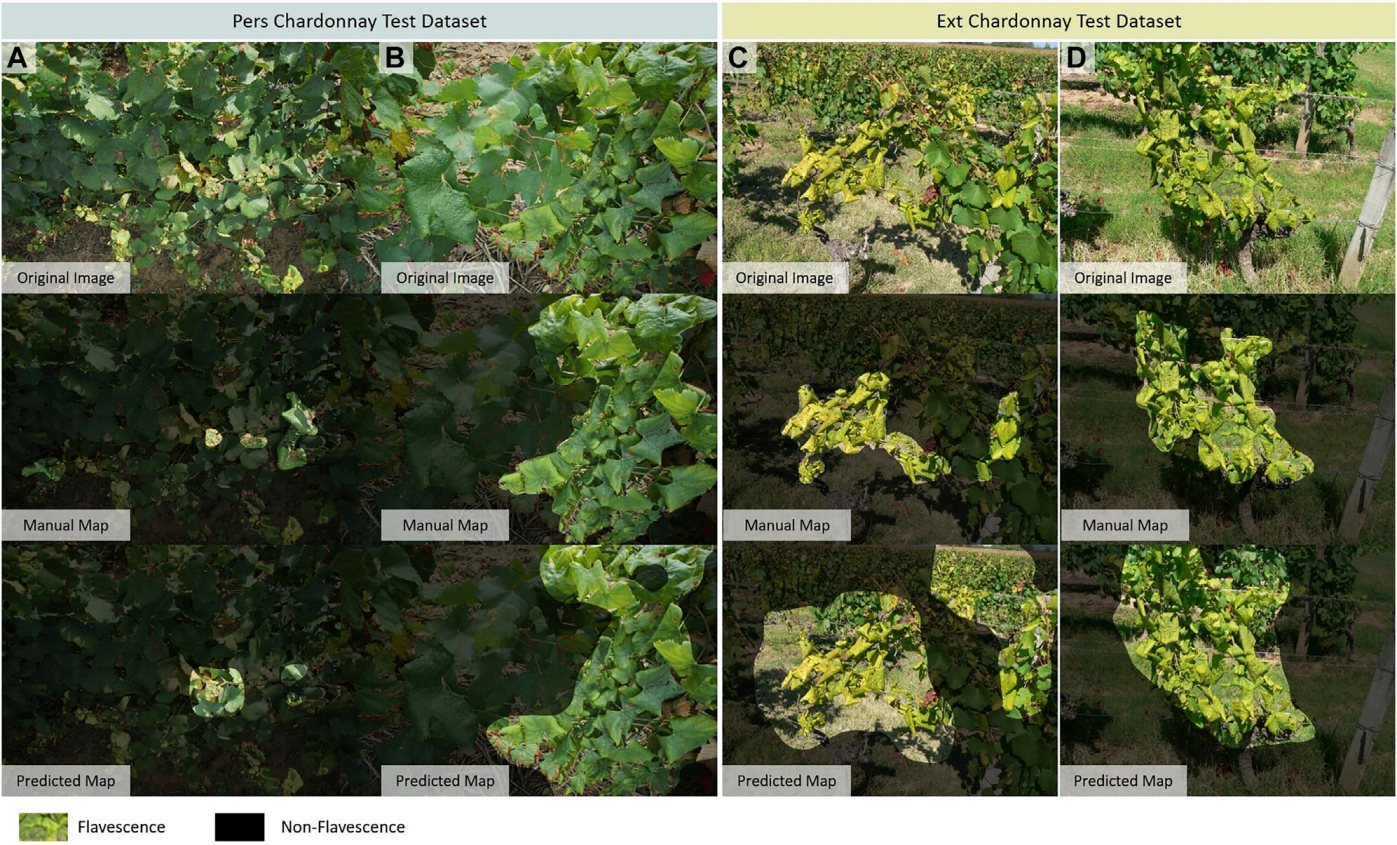
Segmentation maps with minimum and maximum Intersection over Union (IoU) values on Chardonnay datasets. On the *Pers. Chardonnay* dataset, Images **(A)** with the lower IoU value, **(B)** with the higher IoU value. On the *Ext. Chardonnay* dataset, **(C)** with the lower IoU value, **(D)** with the higher IoU value.

**FIGURE 6:**
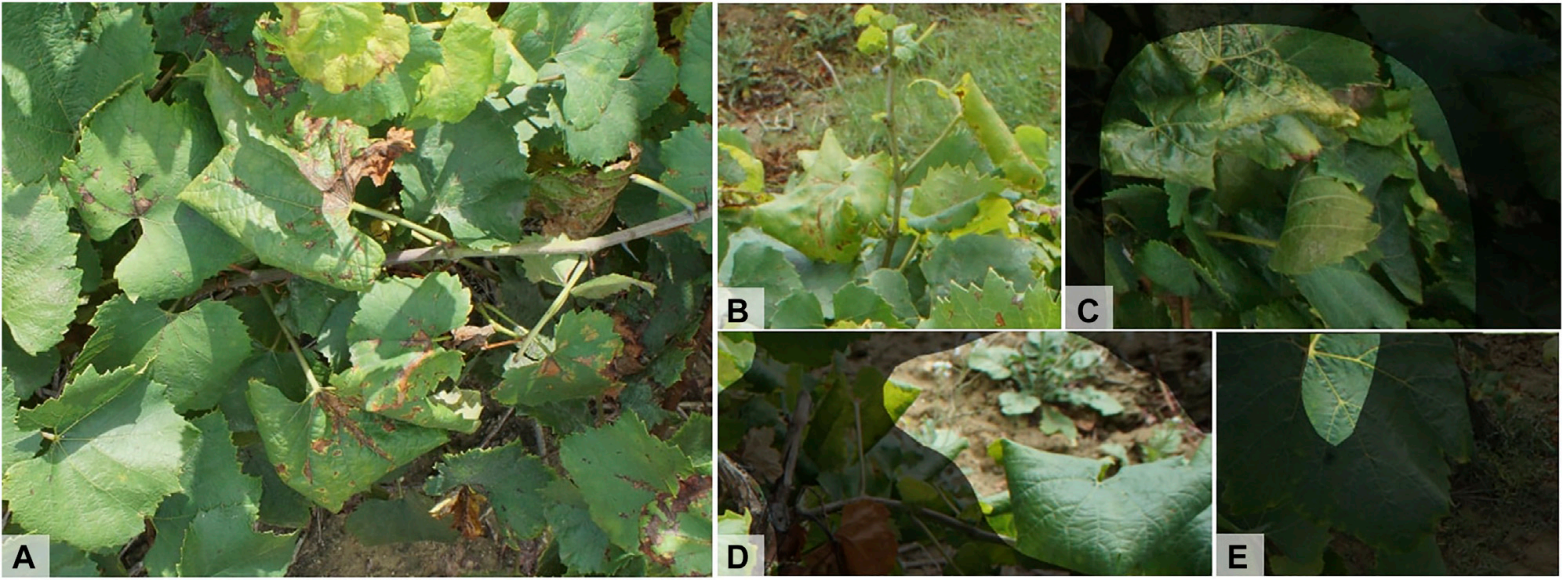
Examples of false predictions from the *Pers. Chardonnay* Test dataset. **(A,B)** Undetected Flavescence dorée (FD) symptoms, **(C,D)** Over-detection close to FD symptomatic areas, **(E)**: Visually unexplainable false FD detection.

Figure 5 presents a set of typical predictions with minimum and maximum IoU values for the two Chardonnay datasets. The minimum IoU prediction on the *Pers. Chardonnay* dataset shows both over- and under-segmentation. However, two of the three leaves with FD symptoms are detected. Overall, we observe a better detection of FD when it is expressed on several leaves or on a branch. When symptoms affect only a few isolated leaves, there is a greater risk of non-detection (6, A and B). Leaves with only the underside visible are also under-detected (Figure 6). Furthermore, even if FD symptoms combined with downy mildew or residues of phytosanitary treatments are mostly detected, we noted a few cases of non-detection in this situation (Figure 6). Confusions with early downy mildew, with an embossed appearance, are present. With the minimum IoU prediction on the *Ext.* Chardonnay dataset, another type of misdetection is related to the background. Grapevines, corn or a hedge in the background can sometimes be partly identified as FD. We also observed a case where another grapevine contaminated with FD in the background was detected. We did not anticipate these two situations during our acquisitions, as we took photos that mainly focused on one grapevine. This image, as well as the maximum IoU prediction on the *Ext. Chardonnay* dataset, also highlight the coarseness of the detection – the FD is properly detected, but the detection mask also includes some of the surrounding ground. Several detections were found, including soil or areas not contaminated with FD, but close to contaminated areas (Figure 6). Finally, false detections that are visually difficult to explain were also found (Figure 6).

As a result, despite the coarseness of the detections and some cases of confusion, the classification model converted to an FCN for large-scale inference is able to detect symptoms of FD on unseen images of Chardonnay grapevines. While the *Ext. Chardonnay* dataset is small, the metrics show that the model is able to generalize on images of the same grape variety, even if they come from another source. The results are very encouraging, as the expression of FD symptoms is slightly different between the two datasets; the leaves are yellower in the *Ext. Chardonnay* images, but they remain quite green in the *Pers. Chardonnay* images. These two expressions, both present in the training set, are well-managed by the model.

### Inference on Several White Grapevine Varieties

**FIGURE 7:**
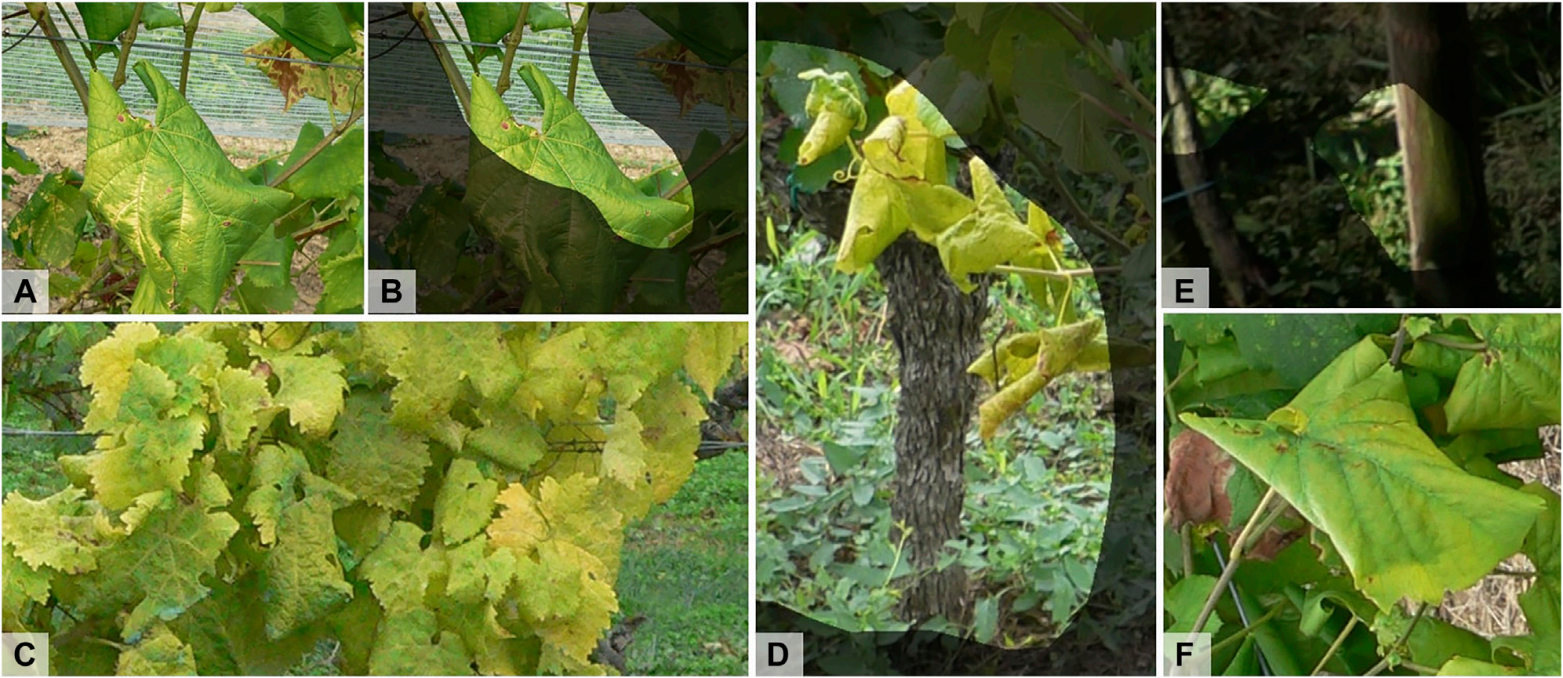
Examples of false predictions from *Ext.* datasets. **(A)** Original image of Exalta leaf with Flavescence dorée (FD) symptoms; **(B)** Prediction associated to image A, only the rolled areas of the leaf are identified as FD; **(C)** Undetected FD symptoms on Ugni-Blanc grapevine variety; **(D)** Over-detection close to FD symptomatic areas; **(E)** False detection on non-grapevine elements; and **(F)** Undetected FD symptoms on Exalta grapevine variety.

**FIGURE 8:**
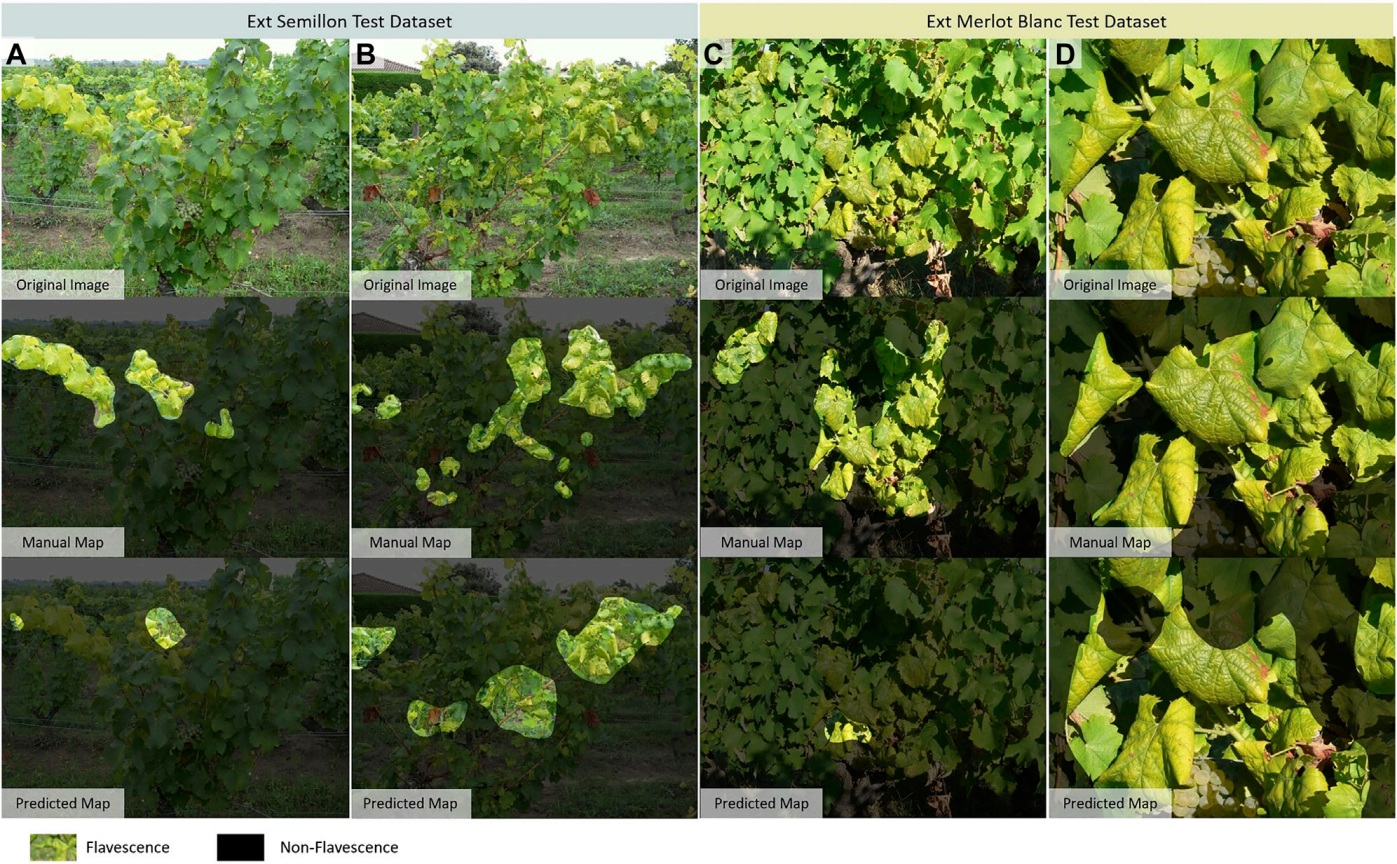
Segmentation maps with minimum and maximum Intersection over Union (IoU) values on two *Ext.* datasets. On the *Ext. Semillon* dataset: Images **(A)** with the lower IoU value, and **(B)** with the higher IoU value. On the *Ext. Merlot Blanc* dataset: **(C)** with the lower IoU value, and **(D)** with the higher IoU value.

Inference using the Chardonnay model on five other white grape varieties gave mixed results (Table 4). For the two Ugni-Blanc datasets, the Chardonnay model is unable to detect FD symptoms: 0% of TPR for *Ext. Ugni-Blanc* dataset and 8.3% of TPR for *Pers. Ugni-Blanc* dataset, with a very poor quality segmentation map for the only TP obtained (IoU value of 0.07). Even when leaves express curling, they are sometimes identified as Non-FD (Figure 7). The same phenomenon was observed on other grape varieties, such as Semillon (Figure 8) or Exalta (Figure 7), which was unexpected given that the model detects Chardonnay symptoms of FD characterized by rolling and significant yellowing (Figure 5). However, one result can be considered positive: the FPR of 2.87% obtained on the *Pers. Ugni-Blanc* dataset is of the same order as the FPR value obtained on the *Pers. Chardonnay* dataset.

**TABLE 4:** Summary of inference results on other grapevine varieties’ datasets, presenting the True Positive Rate (TPR), False Positive Rate (FPR), mean, min and max values of Intersection over Union (IoU).

Test Dataset Name	Number of images	TPR	FPR	IoU mean	IoU min^a^	IoU max
*Pers. Ugni-Blanc*	FD class: 12 Non FD class: 143 Total: 155	8.3% 1 TP/12 P	2.87% 4 FP/139 N	N/A IoU for the only TP: 0.07		
*Ext. Ugni-Blanc*	FD class: 5 Non FD class: none Total: 5	0% 0 TP/5 P	N/A	N/A	N/A	N/A
*Ext. Exalta*	FD class: 12 Non FD class: none Total: 12	83.3% 10 TP/12 P	N/A	0.13	0.02	0.31
*Ext. Merlot Blanc*	FD class: 3 Non FD class: none Total: 3	100% 3 TP/3 P	N/A	0.18	0.01	0.46
*Ext. Riesling*	FD class: 4 Non FD class: none Total: 4	100% 4 TP/4 P	N/A	0.27	0.17	0.39
*Ext. Semillon*	FD class: 5 Non FD class: none Total: 5	100% 5 TP/5 P	N/A	0.14	0.01	0.24

Notes: TP, True Positive; P, Positives; FP, False Positive; N, Negatives.

a IoU min, considering only the True Positive predictions.

The inability of the Chardonnay model to identify FD on Ugni-Blanc could be caused by confusion with downy mildew or nutrients deficiencies. This hypothesis is based on the observation of the predictions for Exalta. In this grape variety, there were more detections on green leaves with heavy rolling. Yellow vein symptoms were classified as Non-FD (Figure 7). This symptom’s expression has not been observed on the visited Chardonnay plots and is therefore absent from the training dataset. For Exalta and Semillon varieties, even if the TPR is high, the segmentation is of poor quality, with many under-detections (Figure 8).

On Riesling and Merlot Blanc varieties, the segmentation is of relatively better quality, with average IoUs of 0.27 and 0.18, respectively. Again, cases of under-detection were noted (Figure 8). One possible explanation could be the difference in resolution between the images used for training and those used for inference. Indeed, in images found in Figures 8C,D, the same leaves were photographed with a more or less close-up view. On the close-up view, FD is detected, while on the farther view, symptomatic leaves are labeled as Non-FD. Furthermore, as observed in the Chardonnay datasets, there is an over-detection in the predictions around areas identified as symptomatic (Figure 7), but there is also over-detection related to the background that is barely interpretable (Figure 7).

In summary, the Chardonnay model does not work to detect FD symptoms on the two Ugni-Blanc datasets. However, the low FPR obtained on the *Pers. Ugni-Blanc* dataset may translate to significant robustness in a real-world application. For the other grape varieties, even if the TPR is high, the average IoUs are very low. In particular, many under-detections were noted. Therefore, the segmentation maps do not have the required quality for an automatic detection tool.

## Discussion

Providing a model with high accuracy for FD symptoms recognition is a first step toward the development of an automatic detection tool, but is not sufficient in itself. Indeed, in agriculture, as in all other fields of application that may have social and environmental impacts, it is necessary to move toward artificial intelligence tools whose results and underlying processes are transparent, understandable and explainable. Relying on opaque results does not provide a sufficient degree of reliability for real-world applications. Wrong predictions in the field could have severe economic consequences for farmers. Without transparency, it would be difficult for farmers to have confidence in new AI-driven tools. Explainable AI refers to all the methods and techniques put in place to allow humans to understand the decisions and results produced by AI systems, thus avoiding a black box effect (Holzinger et al., 2019; Doran et al., 2018; Arrieta et al., 2020).

Here, we take a step toward greater transparency by increasing the interpretability of our system regarding two aspects: 1) the multi-varietal application capability of the model trained only on Chardonnay FD symptoms, and 2) the general robustness of the model. For this purpose, we use two visualization techniques. The first one, Guided Gradient-weighted Class Activation Mapping (GG-CAM, Selvaraju et al., 2019),^6^ provides a finely detailed and discriminative view of class activations. This allows us to observe which characteristics of an image are used to predict a targeted class. The second visualization technique, Uniform Manifold Approximation and Projection (UMAP, McInnes et al., 2018a),^7^ is a a dimensionality reduction algorithm that preserves the global structure of the projected data. This allows us to visualize the spatial arrangement of the image embeddings inferred by the model. Here, we use UMAP to observe the closeness of the embeddings according to their class and grape variety.

To improve the readability of this analysis, we used a subset of our evaluation datasets to create several visualizations. This subset contains 360 patches extracted from the images within the six evaluation datasets. Specifically, 30 patches were extracted for each of the following 12 categories: *Pers. Chardonnay*, Healthy; *Pers. Chardonnay*, Downy Mildew; *Pers. Chardonnay*, Deficiencies; *Pers. Chardonnay*, FD; *Ext. Chardonnay*, FD; *Pers. Ugni-Blanc*, Healthy; *Pers. Ugni-Blanc*, FD; *Ext. Ugni-Blanc*, FD; *Ext. Exalta*, FD; *Ext. Merlot Blanc*, FD; *Ext. Riesling*, FD; *Ext. Semillon*, FD.

### Multi-Varietal Application

**FIGURE 9:**
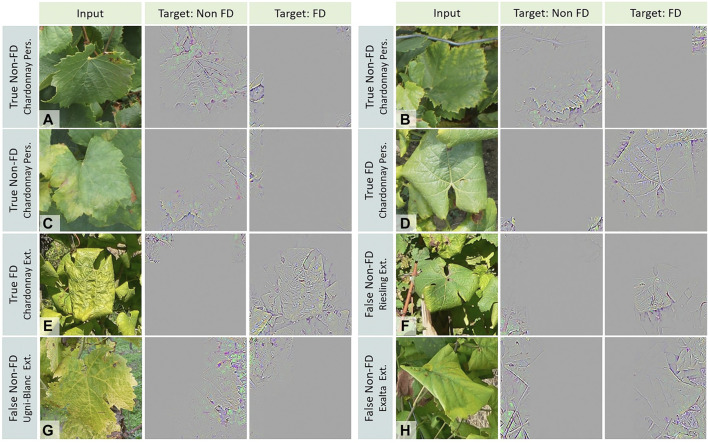
Illustration of representative features from Guided Grad-CAM that are used by the model to predict the Flavescence dorée (FD) and Non-FD classes. (**A–C)**: Non-FD images, **(D–H)**: FD images. The predicted label and the source dataset are given next to each image.

We expect that the need for FD detection will appear for many grape varieties. If images have to be collected on all the targeted grape varieties, the task would become increasingly challenging and expensive. Our model, trained on a single symptomatic white grape variety, resulted in mixed performances when tested on other varieties. By applying GG-CAM to our images, the characteristics typically used by the model to associate FD and Non-FD classes are highlighted (Figure 9). For both classes, leaf morphology seems to be important. For the Non-FD class, it is mainly the leaf teeth that stand out (Figures 9A–C,G), but the orientation of the veins also appears to be relevant (Figure 9A). For the FD class, morphologically, it is mainly the curl, the sinus shape and the vein orientation that emerge (Figures 9D–F,H). Leaf texture also seems to be a discriminating feature, as an embossed, scaly aspect of the leaves appears (Figures 9E,F,H). Color is also a decisive feature. For the FD class there is a yellow dominance, whereas for the Non-FD class green and purple spots are frequently seen. Canes and petioles are mostly correctly associated with the Non-FD class (Figures 9A,F,H). The model’s discriminative leaf features spotted through GG-Cam are of interest since they reflect the characteristics used by experts in the field for their diagnosis.

Such general class feature descriptions provide a better understanding about FD false negative predictions across the different grape varieties. The Ugni-Blanc, which here expresses little curling, seems to be associated with the Non-FD class because of the toothed aspect of the leaf, which remains despite FD contamination (Figure 9G). In some cases, including those showing partial curling, both classes have strong activations (Figure 9F,H), but the Non-FD class is predicted by the model. This problem may have been accentuated here by the size of the image patch as well as by the core size of the average pooling layer. Some FD symptoms do not appear in the GG-CAM maps, including vein discoloration and FD-symptomatic leaves without curling.

**FIGURE 10:**
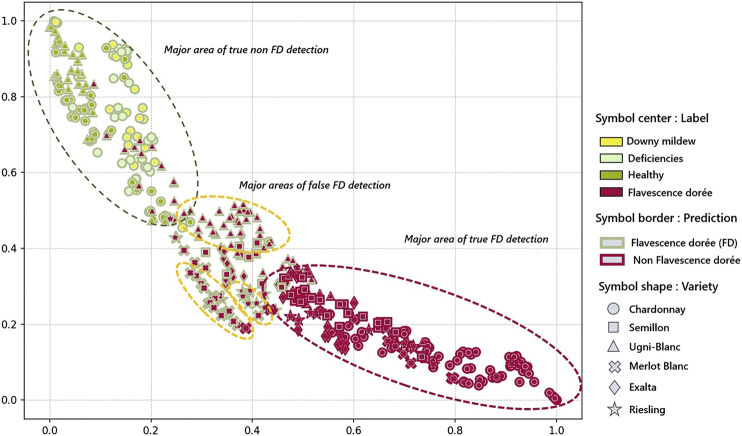
Grape varieties in the model space: UMAP visualization of the embeddings based on an arbitrarily-set seed.

The unawareness of these symptoms, which are very common for some grape varieties, is also revealed through UMAP visualization (Figure 10). Indeed, in this visualization, two clusters are found at each end: the true predictions of Non-FD and FD. In the Non-FD cluster, healthy Ugni-Blanc and healthy Chardonnay share the same space. Downy mildew and deficiencies embeddings are also quite close. At one end of this cluster, we observe proximity to the true Non-FD positives that correspond to Chardonnay deficiencies and downy mildew patches with false Non-FD negatives of Ugni-Blanc.

In the true FD prediction cluster, all of the Chardonnay patches from the *Pers.* and *Ext.* datasets are found with the majority grouped at the 0.8:1 end of the *x*-axis. Elsewhere in the cluster, many Semillon, some Exalta, Riesling, Merlot Blanc and, to a lesser extent, Ugni-Blanc can be found. In between our two main clusters of true predictions is a zone of false negative FD embeddings. In this area, there are micro-clusters based on grape variety. These are certainly influenced by the closeness of the expressed symptoms among the same grape variety, but potentially also by having images from the same source. Despite this possible bias, the UMAP visualization confirms the hypothesis that the model fails to detect FD symptoms on Ugni-Blanc because of their closeness to deficiencies and to downy mildew symptoms. For false detections on the other grape varieties, a greater distance is observed from the cluster of good Non-FD predictions, which expresses the model’s ambiguity toward symptom expressions not directly seen during training. To improve this model and make it multi-varietal, new acquisitions would be necessary to include images with grapevines presenting symptoms with intermediate curling intensities and vein colorings.

### Toward a Robust Detection Model

Although the results obtained on Chardonnay are promising, further acquisitions are needed to increase our model’s reliability. Indeed, even for only FD on a given grape variety, symptoms may be expressed differently from one year to another. In addition, the general phytosanitary situation of a field can be very different from one year to another. Since FD is a late-season disease, grape leaves may already be damaged when FD symptoms appear. Acquisitions over several years will provide the needed data on the co-occurrence of phytosanitary problems.

**FIGURE 11:**
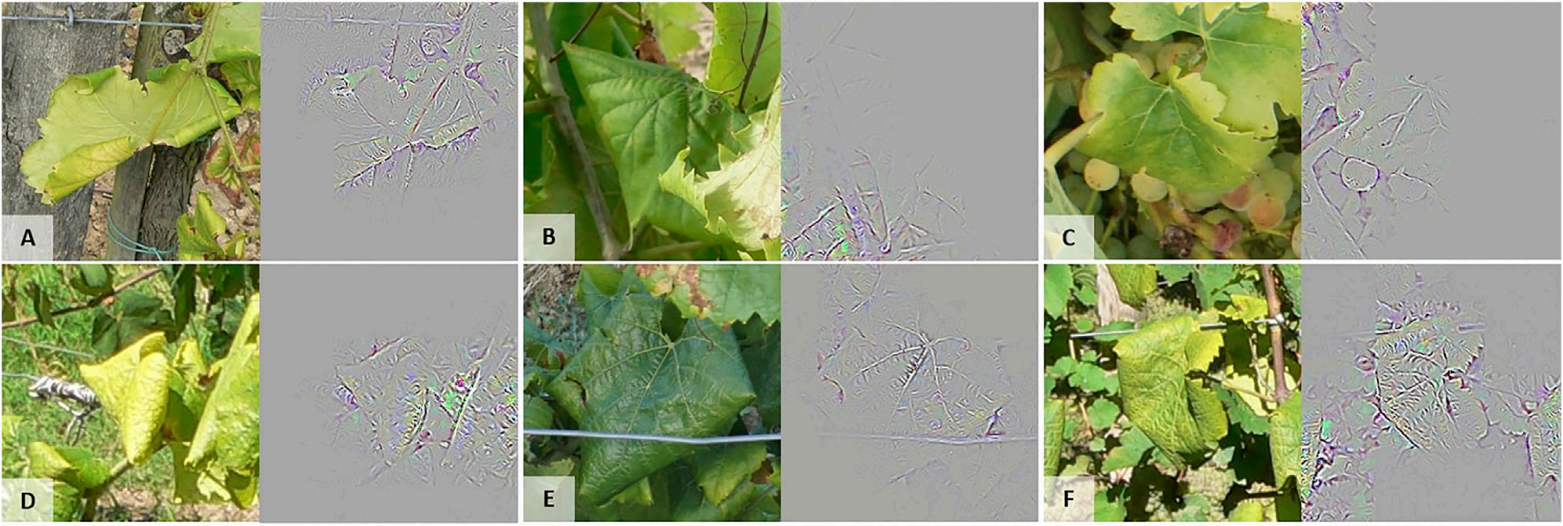
Illustration of irrelevant features from GG-CAM that are used by the model to predict the Flavescence dorée (FD) class. **(A)** Trellising wire, petioles, canes, soil and rolled shapes, **(B)** Canes, **(C)** Canes and grapes, **(D)** Trellising wire and triangular shadow shape, **(E)** Trellising wire and triangular shadow shape, and **(F)** Trellising wire and soil.

The weaknesses of our model can also be targeted using the feature maps produced with GG-CAM (Figure 11). Indeed, some elements of the image stand out for the FD target when they should not, such as the trellising wire (Figures 11A,D,E,F), the soil (Figures 11A,D,F and 9H), some shadows or leaf overlays with triangular or rolled shapes (Figures 11D and 9A,H), as well as, in rare cases, grapes, petioles or canes (Figures 11A–C). To overcome this, targeted acquisitions to increase training image diversity could be conducted. Intervention at the model level could also be performed, e.g. by adding the annotation mask as an extra channel during training to act as an attention map, or with explanatory interactive learning, as in Schramowski et al. (2020) for plant phenotyping. These techniques would allow us to ensure that the predictions obtained are based on relevant features.

## Conclusion

In this study, we sought to automatically pre-identify FD symptoms on several white grape varieties. We trained a CNN model and turned it into an FCN so that it produced segmentation of areas expressing FD symptoms. In the training dataset, all symptomatic annotations were based on Chardonnay grapevines. During evaluation, this model obtained high accuracy on Chardonnay images, both on the personal and on the external datasets. Using the GG-CAM visualization technique, weaknesses in the model were identified, such as sensitivity to background or to trellising elements. Further targeted acquisitions and re-training could solve this problem, which is highly encouraging. A larger external dataset obtained over several growing seasons would nevertheless be necessary to ensure that this model is indeed operational on Chardonnay grapevines.

For the other grape varieties, the results obtained were more nuanced, and showed how different symptoms’ expressiveness could be problematic for FD pre-identification. As expected, the model performed poorly for the grape variety expressing the least amount of curling, i.e., Ugni-Blanc. We were able to confirm this hypothesis using GG-CAM visualizations. The embeddings extracted through convolutions for FD-contaminated Ugni-Blanc leaves are close to those of leaves affected by downy mildew and deficiencies. For the other grape varieties, the performance is mixed, with very high prediction rates. For a model trained with a grape variety expressing a lot of curling, good results were achieved on grape varieties expressing curling to a lesser extent. This underlines the model’s relative flexibility and validates the idea that the development of a multi-varietal model is achievable.

Our next steps are to develop a comprehensive overview of the various FD symptoms and to include them in a multi-varietal dataset. Appropriate precautions will be undertaken regarding all other diseases that may express yellowing and curling. Capturing images from these misleading negatives would help to establish a more accurate boundary between FD and Non-FD images. Finally, going back and forth between model training and field testing will allow us to develop a robust detection tool. Using visualization techniques, as we have done in this study, will help to provide a more complete model analysis, and thus help to achieve the reliability needed for in-field applications.

## Data Availability

The data analyzed in this study is subject to the following licenses/restrictions: non-commercial use only. Requests to access these datasets should be directed to JB, justine.boulent@usherbrooke.ca

## Footnotes

1 Available online: https://flavescencedoree.jimdofree.com/

2
https://pytorch.org/tutorials/beginner/transfer_learning_tutorial.html

3
https://augmentor.readthedocs.io/en/master/

4
https://docs.fast.ai/

5
https://github.com/plstcharles/thelper

6 Python library used: Captum (Kokhlikyan et al., 2019)

7 The Python library used here: UMAP (McInnes et al., 2018b)
